# Ageing‐related modification of sleep and breathing in orexin‐knockout narcoleptic mice

**DOI:** 10.1111/jsr.14287

**Published:** 2024-07-20

**Authors:** Stefano Bastianini, Sara Alvente, Chiara Berteotti, Viviana Lo Martire, Gabriele Matteoli, Elena Miglioranza, Alessandro Silvani, Giovanna Zoccoli

**Affiliations:** ^1^ Laboratory of Physiological Regulation in Sleeping Mice (PRISM), Department of Biomedical and Neuromotor Sciences Alma Mater Studiorum – University of Bologna Bologna Italy

**Keywords:** apnea, cataplexy, elderly, hypnic, rodent

## Abstract

Narcolepsy type‐1 (NT1) is a lifelong sleep disease, characterised by impairment of the orexinergic system, with a typical onset during adolescence and young adulthood. Since the wake–sleep cycle physiologically changes with ageing, this study aims to compare sleep patterns between orexin‐knockout (KO) and wild type (WT) control mice at different ages. Four groups of age‐matched female KO and WT mice (16 weeks of age: 8 KO‐YO and 9 WT‐YO mice; 87 weeks of age: 13 KO‐OLD and 12 WT‐OLD mice) were implanted with electrodes for discriminating wakefulness, rapid‐eye‐movement sleep (REMS), and non‐REMS (NREMS). Mice were recorded for 48 h in their home cages and for 7 more hours into a plethysmographic chamber to characterise their sleep‐breathing pattern. Regardless of orexin deficiency, OLD mice spent less time awake and had fragmentation of this behavioural state showing more bouts of shorter length than YO mice. OLD mice also had more NREMS bouts and less frequent NREMS apneas than YO mice. Regardless of age, KO mice showed cataplexy‐like episodes and shorter REMS latency than WT controls and had a faster breathing rate and an increased minute ventilation during REMS. KO mice also had more wakefulness, NREMS and REMS bouts, and a shorter mean length of wakefulness bouts than WT controls. Our experiment indicated that the lack of orexins as well as ageing importantly modulate the sleep and breathing phenotype in mice. The narcoleptic phenotype caused by orexin deficiency in female mice was substantially preserved with ageing.

## 1 INTRODUCTION

Narcolepsy type‐1 (NT1) is a rare sleep disorder linked to the functional loss of hypothalamic neurons producing orexins (also known as hypocretins) (Peyron et al., 2000; Seifinejad et al., 2023). Narcolepsy type‐1 starts most often during childhood or adolescence and persists lifelong thereafter (Kovalská et al., 2016; Lividini et al., 2021). The natural history (i.e. the disease progression in an individual over time, in the absence of treatment) of narcolepsy type‐1 is still incompletely understood, particularly throughout ageing. This uncertainty also concerns age‐related changes in excessive daytime sleepiness and cataplexy, which are cardinal symptoms and signs of narcolepsy type‐1 (Furuta et al., 2001; Lamphere et al., 1989; Lividini et al., 2021). Fewer sleep‐onset rapid‐eye‐movement sleep (REMS) periods have been reported in patients with narcolepsy type‐1 with increasing age (Lividini et al., 2021), whereas physiological ageing has a minimal association with decreased REMS latency (Li et al., 2018). On the other hand, senior patients with narcolepsy type‐1 more often report disturbed night‐time sleep (Lividini et al., 2021) and have a lower sleep efficiency (Lamphere et al., 1989) and more time spent in stage N1 sleep (Lividini et al., 2021) than younger patients. However, similar changes also characterise healthy ageing (Li et al., 2018). Obstructive sleep apneas occur frequently in adult patients with narcolepsy type‐1 (Pataka et al., 2012; Pizza et al., 2013; Sansa et al., 2010), particularly during REMS (Hoshino et al., 2019), and their occurrence rate is higher in senior and middle‐aged adults than in children and adolescent patients (Lividini et al., 2021). Nevertheless, this conclusion may be confounded by the age‐related increase in the prevalence of obstructive sleep apnea that also occurs in the general population (Senaratna et al., 2017).

Studies on animal models of narcolepsy type‐1 and age‐matched control animals may help to clarify the biological bases of the natural history of narcolepsy type‐1 with full control of confounding effects such as those related to physiological ageing. In particular, the study of orexin knock‐out (KO) mice, which recapitulates the human narcolepsy type‐1 phenotype (Chemelli et al., 1999), may help to focus on the specific contribution of orexin deficiency on the narcolepsy phenotype across the lifespan, which may be especially relevant in light of the recent development of orexin receptor agonists for the treatment of NT1 (Dauvilliers et al., 2023).

We aimed to investigate the natural history of the narcolepsy type‐1 phenotype during ageing in orexin KO mice, focussing on alterations in wake–sleep states, cataplexy, and breathing compared with age‐matched wild‐type (WT) controls.

## 2 METHODS

### 2.1 Ethical approval

The study protocol complied with the EU Directive 2010/63/EU for animal experiments and was approved by the Committee on the Ethics of Animal Experiments of the Italian Ministry of Health (Prot. no. 667/2019). The experiments were carried out according to the guidelines of the animal welfare committee of the University of Bologna, Italy, and ARRIVE guidelines. All efforts were made to minimise animal suffering.

### 2.2 Mice and experimental protocol

Four groups of female mice were included in the present experiment: young‐adult orexin KO mice (KO‐YO, *n* = 8), young‐adult WT mice (WT‐YO, *n* = 9), old orexin KO mice (KO‐OLD, *n* = 13), and old WT mice (WT‐OLD, *n* = 12). WT‐YO and WT‐OLD mice were littermates of KO‐YO and KO‐OLD mice, respectively. All mice were congenic (*N* > 10) with a C57BL/6J genetic background. The age at surgery of the mice in different groups was 15.6 ± 0.2 and 15.8 ± 0.1 weeks for KO‐YO and WT‐YO, respectively, and 86.9 ± 1.1 and 85.7 ± 0.1 weeks for KO‐OLD and WT‐OLD, respectively. KO‐YO and WT‐YO were age‐matched (*p* = 0.328, *t*‐test) and so were KO‐OLD and WT‐OLD (*p* = 0.298, *t*‐test). Between 13 and 26 weeks of age, C57BL/6J mice are considered mature adults approximately comparable to human subjects aged 20–30 years. Between 78 and 104 weeks of age, C57BL/6J mice present senescent changes in almost all biomarkers and can be approximately compared with human subjects aged 56–69 years (Flurkey et al., 2007). All animals were born and raised in the animal facility of the Department of Biomedical and Neuromotor Sciences at the University of Bologna, Italy, with 25°C ambient temperature, food and water ad libitum, and 12:12 light–dark cycle (LD, with light on at Zeitgeber Time, ZT0: 9:00). Each mouse was anaesthetised (1.8–2.4% isoflurane in O_2_), injected with analgesic (Carprofen 0.1 mg; Pfizer Italy, Latina, Italy), and then implanted with a pair of Teflon‐coated stainless‐steel screws (Cooner Wire, Chatsworth, CA, USA) in contact with the dura mater in the frontal and parietal bones to obtain a differential electroencephalographic (EEG) signal. A second pair of electrodes was inserted bilaterally in the nuchal muscles to obtain a differential electromyographic (EMG) signal. All electrodes were connected to a miniature custom‐built socket, which was cemented to the skull with stainless‐steel anchor screws (2.4 mm length, Plastics One, Roanoke, VA, USA), dental cement (Rely X Unicem, 3 M ESPE, Segrate, Milano, Italy), and dental acrylic (Respal NF, SPD, Mulazzano, Italy) (Bastianini et al., 2021). Antibiotic therapy (3750 I.U. of 250000 I.U./mL benzathine benzylpenicillin and 1.5 mg of 100 mg/mL dihydrostreptomycin sulphate, strong Rubrocillin®, MSD Animal Health s.r.l., Milan, Italy) was injected subcutaneously at the end of the surgery.

After the surgery, the mice were housed individually and allowed 5–7 days to recover fully (Berteotti et al., 2021). Then, the mice were briefly anaesthetised (1.8%–2.4% isoflurane in O_2_) to connect the electrodes to a recording cable which, in turn, was plugged into a rotating swivel (SL2 + 2C/SB, Plastics One, Roanoke, VA, USA) and a balanced cable suspensor allowing unhindered movements to the mice (Bastianini et al., 2021). From then on, milk chocolate was added to the cage to increase the occurrence of cataplectic events in narcoleptic mice (Oishi et al., 2013). After 2 more days habituation to the experimental setup (Berteotti et al., 2021), simultaneous recordings of EEG and EMG signals were performed continuously for 48 h on mice undisturbed and freely behaving in their own cages with the ambient temperature set at 25°C, food and water ad libitum, and a LD cycle of 12:12 h. The EEG and EMG signals were amplified, filtered (EEG: 0.3–100 Hz; EMG: 100–1000 Hz; 7P511J amplifiers, Grass, West Warwick, RI, USA), sampled at 1024 Hz, and down‐sampled at 128 Hz for data storage. The EEG and EMG amplifier gains were adjusted for each mouse to avoid signal saturation (Bastianini et al., 2014).

Five to seven days later, at lights on, the mice were individually placed inside a whole‐body plethysmograph (WBP, model PLY4223, Buxco, Wilmington, NC, USA) to simultaneously record EEG, EMG, and breathing for 6 h (Berteotti et al., 2020). Mice were habituated to this specific recording setup for 1 h before starting the recordings (i.e. from ZT0 to ZT1). Recordings in the whole‐body plethysmograph then lasted from ZT1 to ZT7. The respiratory signal was derived from the differential pressure between the mouse chamber and a second reference chamber, measured with a high‐precision differential pressure transducer (DP103‐06 + CD223 digital transducer indicator; Validyne Engineering, Northridge, CA, USA). The differential pressure, chamber humidity, and temperature were continuously recorded, digitised, and stored at 128, 4, and 4 Hz, respectively. The whole‐body plethysmograph chamber was continuously flushed with compressed air at 1.5 L/min, and the system was calibrated dynamically with a 100 μL micro‐syringe (Hamilton, Reno, NV, USA) at the end of each recording (Bastianini et al., 2017).

### 2.3 Data analysis

Data analysis was performed with custom‐made MatLab (Mathworks, Natick, MA, USA) scripts. Sleep scoring during the 48 h of home‐cage recordings was performed automatically on 4 s epochs based on EEG and EMG signals with a validated algorithm (SCOPRISM) (Bastianini et al., 2014). The analysis of sleep architecture was performed on 48 h home‐cage recordings with a threshold of 12 s (i.e. 3 consecutive 4 s epochs) for wake–sleep episode (bout) length (Bastianini et al., 2011). Cataplexy‐like episodes (CLE) were identified following consensus criteria (Scammell et al., 2009) as sleep‐onset REMS episodes after a sustained episode of wakefulness. Each detected CLE was visually checked by an expert investigator based on raw EEG and EMG recordings. A sleep fragmentation index (SFI) was calculated as the number of brief awakenings (episodes lasting less than 120 s; Bastianini et al., 2015) over the total sleep time. Spectral analysis of the EEG signal during home‐cage recordings was performed on artefact‐free 4 s epochs using the discrete Fourier transform. The slow wave activity (SWA) in NREMS (δ frequency band) and the EEG power spectrum in NREMS and REMS were analysed, focussing on the frequency corresponding to the spectrum peak in each state (Bastianini et al., 2021). EEG spectral power and SWA were expressed as the percentage of the total EEG power in each state because the EEG signal was not calibrated before each recording.

Sleep scoring during the 6 h sessions inside the whole‐body plethysmograph was performed on 4 s epochs with visual inspection of raw EEG and EMG recordings by trained investigators using published criteria consistent with those applied by SCOPRISM (Bastianini et al., 2014). SCOPRISM was not used to score sleep during the 6 h recordings inside the whole‐body plethysmograph because it was validated for recordings longer than 24 h, which may be relevant because the algorithm sets EMG thresholds based on the empirical distribution of EMG data (Bastianini et al., 2014). Wakefulness was scored when the EMG tone was high and the EEG was at a low voltage. Non‐rapid‐eye‐movement sleep (NREMS) was scored when the EMG tone was lower than in wakefulness and the EEG was at a high voltage with prominent δ (0.5–4 Hz) frequency components. REMS was scored when the EMG indicated muscle atonia with occasional muscle twitches and the EEG was at a low voltage with predominant θ (6–9 Hz) frequency components. Epochs with signals that were borderline between two different states were scored as “undetermined” (Bastianini et al., 2014).

The ventilatory period (i.e. the interval between successive breaths, VP), tidal volume (TV, expressed per gram body weight), and minute ventilation (MV, expressed per gram body weight) were calculated from the respiratory signal recorded inside the whole‐body plethysmograph for each mouse in each sleep state. Augmented breaths (sighs) during NREMS were detected as breaths with TV >3 times the average TV in NREMS. Breathing pauses (apneas) during NREMS and REMS were detected as breaths with VP >3 times the average VP in NREMS or REMS, respectively (Bastianini et al., 2019). Each individual sigh and apnea was visually checked based on respiratory recordings by expert investigators to exclude artefacts.

### 2.4 Statistics

Data were first tested for normal distribution using the Shapiro–Wilk test. When the normality assumption was rejected, we first analysed data with the Kruskal‐Wallis test and, in case the null hypothesis was rejected, we applied the Mann–Whitney test with the false discovery rate correction (Curran‐Everett, 2000) for the following pre‐planned comparisons: KO‐YO versus WT‐YO, KO‐YO versus KO‐OLD, WT‐YO versus WT‐OLD, WT‐OLD versus KO‐OLD, YO versus OLD (to focus on the overall effect of age), and WT versus KO (to focus on the overall effect of genotype). The normality assumption was rejected for the EEG power spectrum peak, SFI and for all the whole‐body plethysmograph variables analysed. When the normality assumption was not rejected, we performed two‐ or three‐way ANOVAs with genotype (2 levels), age (2 levels), and LD period (2 levels) as fixed factors. Simple effects were tested with Sidak's correction for multiple comparisons in case of significant interactions between or among factors. An independent‐sample *t*‐test was used to analyse the differences in CLE between KO‐YO and KO‐OLD mice. Data were analysed with GraphPad Prism (version 9) and are reported as mean ± SEM or as median and range depending on whether normality was assumed or not. Significance was set at *p* < 0.05. A principal components analysis (PCA) was performed on EEG, sleep, and breathing features of all the mice included in this study to reduce the dimensionality of our dataset while preserving the global variability among experimental groups. PCA was performed on 31 standardised variables (Table S2), excluding EEG spectral powers at multiples of the fundamental frequency, which would have inflated the number of variables in the PCA with limited added information, and MV values, which are the products of TV and VP.

## 3 RESULTS

### 3.1 Body weight

Body weight at surgery was 21.0 ± 0.4 g in KO‐YO, 20.2 ± 0.5 g in WT‐YO, 25.7 ± 0.9 g in KO‐OLD, and 26.6 ± 0.6 g in WT‐OLD mice. OLD mice were significantly heavier than YO mice (2‐way ANOVA) with no significant differences between genotypes and no significant genotype × age interaction.

### 3.2 Sleep architecture and cataplexy during home‐cage recordings

**FIGURE 1:**
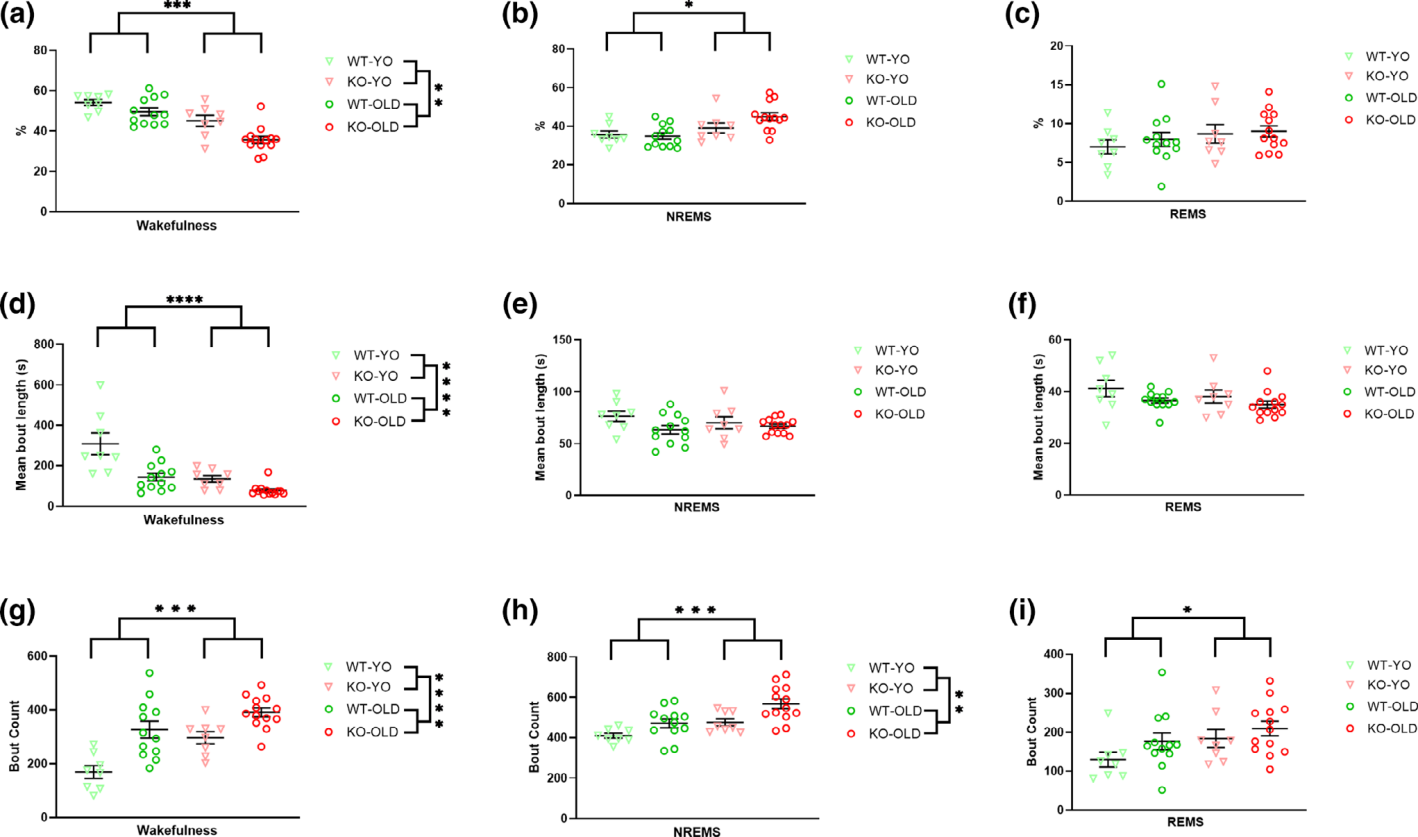
Wake–sleep structure during home‐cage recordings. (a–c) Percentage of time spent in wakefulness, non‐rapid‐eye‐movement sleep (NREMS), and rapid‐eye‐movement sleep (REMS), respectively, by OLD and young (YO) orexin‐knockout (KO) and wild‐type (WT) control mice. (d–f) Mean episode length during wakefulness, NREMS, and REMS, respectively. Data are reported as mean ± SEM, with symbols representing data in individual mice. *, **, ***, and **** *p* < 0.05, *p* < 0.01, *p* < 0.001, and *p* < 0.0001, respectively, for the main effect of genotype or age (2‐way ANOVA)

**FIGURE 2:**
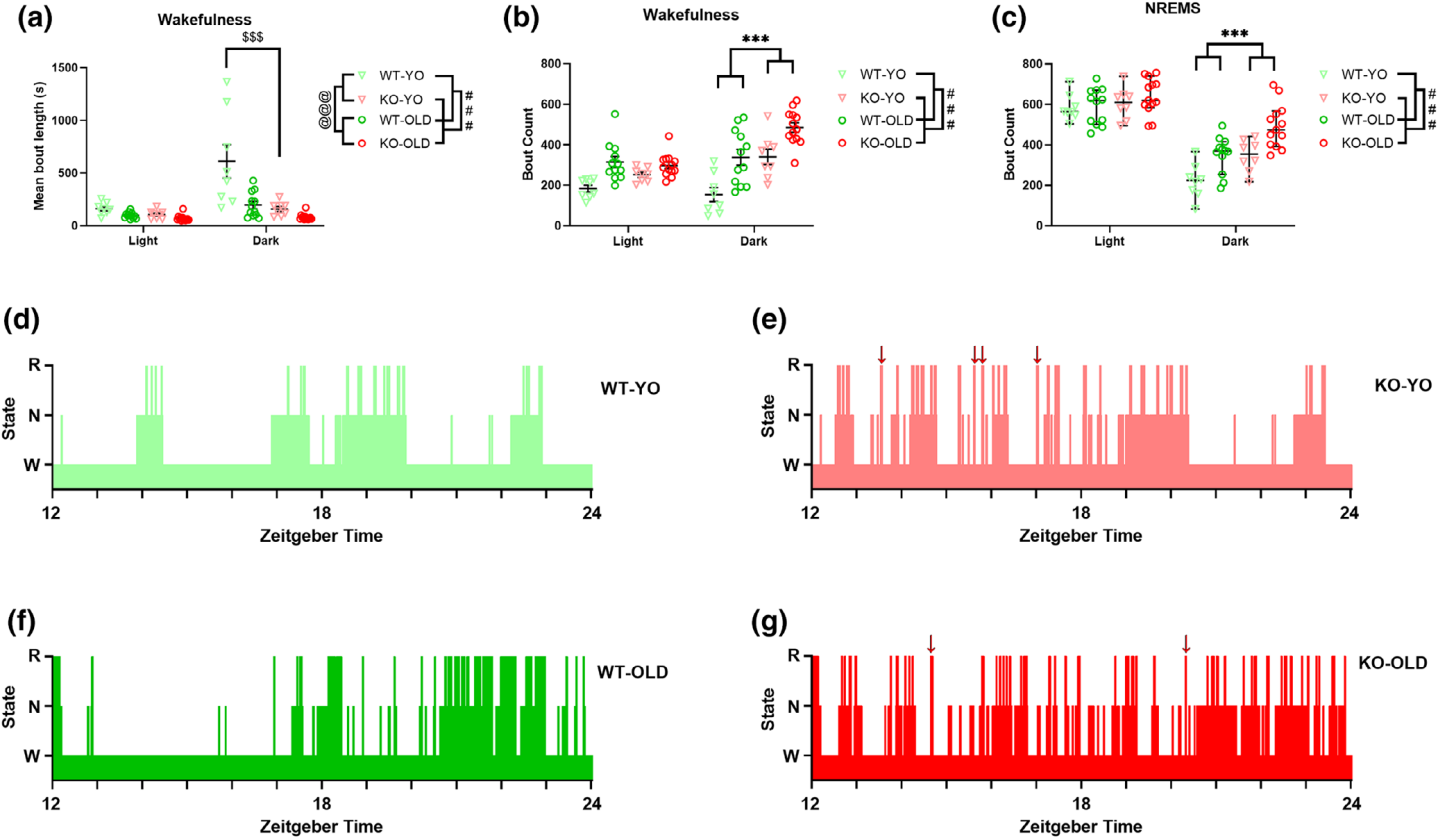
Wake–sleep structure as a function of the light–dark period in home‐cage recordings. (a, b) mean length and number of wakefulness episodes. (c) Number of non‐rapid‐eye‐movement sleep (NREMS) episodes, as a function of the light (resting) and the dark (active) period in OLD and young (YO) orexin‐knockout (KO) and wild‐type (WT) control mice. Data are reported as mean ± SEM, with symbols representing data in individual mice. ^###^ and ^@@@^ *p* < 0.001, main effect of genotype or age (3‐way ANOVA), respectively. ***p < 0.001, post‐hoc corrected comparison between KO and WT in the dark period, with significant photoperiod × genotype interaction at 3‐way ANOVA. ^$$$^ *p* < 0.001 for the post‐hoc corrected comparison between KO‐YO and WT‐YO, with significant age × genotype × photoperiod interaction or age × genotype interaction at 3‐way ANOVA. (d–g) Representative 12 h hypnograms (dark period, from Zeitgeber 12 to 24) of WT‐YO, KO‐YO, WT‐OLD and KO‐OLD, respectively. N, NREMS; R, REMS; W, Wakefulness. Red arrows highlight cataplexy‐like episodes in KO mice. KO, orexin‐knockout; WT, wild‐type; YO, young.

Figure 1 shows the total (%) time, the bout number, and the mean bout length of wakefulness, NREMS and REMS during home‐cage recordings. The percentage of recording time scored as undetermined state was similar between the two genotypes and increased significantly with age independently of genotype (4.1% ± 0.7% vs. 8.6% ± 0.9% in YO vs. OLD mice, respectively). Overall, KO mice spent significantly less time awake (Figure 1a) and the fragmentation of wakefulness (i.e. increased number of wakefulness bouts with shorter mean bout length, Figure 1d,g) than WT mice (two‐way ANOVA, genotype main effect). KO mice also spent more time in NREMS (Figure 1b) due to an increased number of NREMS bouts (Figure 1h) than WT (two‐way ANOVA, genotype main effect). The difference between KO and WT mice in the number of both wakefulness and NREMS bouts was significantly accentuated during the dark (active) period (post‐hoc analyses with significant LD × genotype interaction) as shown in Figure 2a,b. KO mice exhibited a similar total amount and episode length of REMS compared with WT controls (Figure 1c,f) but an increased number of REMS bouts (two‐way ANOVA, genotype main effect, Figure 1i). Finally, SFI did not significantly differ between genotypes (median [range] values: 45.0 [31.0] eps/h vs. 45.6 [63.6] eps/h, for KO and WT, respectively).

Ageing (ANOVA main effect) significantly modulated both wakefulness (total amount, number and length of episodes) and NREMS (number of episodes). In particular, OLD mice exhibited less time awake (Figure 1a) and fragmentation of wakefulness (i.e. increased number of wakefulness bouts with shorter mean bout length, Figure 1d,g) compared with YO mice. Moreover, OLD mice showed an increased number of NREMS episodes compared with YO mice (Figure 1h). Finally, SFI was significantly higher in OLD (48.2 [58.5] eps/h) than in YO mice (41.2 [34.4] eps/h, post‐hoc analyses with significant Kruskal‐Wallis test).

The only significant interaction involving age and genotype effects at three‐way ANOVA regarded wakefulness bout length, which at post‐hoc analysis was significantly shorter in KO‐YO than in WT‐YO during the dark period. No significant difference in wakefulness bout length occurred between WT‐OLD and KO‐OLD, indicating that the mean length of wakefulness bouts was affected by age in WT but not in KO mice (Figure 2a). Figure 2d–f shows representative hypnograms during the dark period of the four experimental groups under study.

As expected (Bastianini et al., 2011), CLE occurred only in KO mice and REMS latency was significantly lower in KO than in WT (Table 1). ANOVA did not reveal any significant main effect of ageing or ageing × genotype interaction on REMS latency. The CLE occurrence rate did not differ significantly between KO‐YO and KO‐OLD mice (*p* = 0.132, *t*‐test).

**TABLE 1:** REM sleep latency and cataplexy‐like episode occurrence rate during home‐cage recordings.

Experimental group		REMS latency (s)		CLE (n/ 24 h)
Genotype	Age	Light	Dark	
WT	YO	603 ± 56	531 ± 62	0
	OLD	600 ± 50	463 ± 72	0
KO*	YO	448 ± 52	281 ± 62	27 ± 4
	OLD	533 ± 45	283 ± 29	17 ± 4

*Note*: Rapid‐eye‐movement sleep (REMS) latency during the light and dark period and cataplexy‐like episodes (CLE) in young (YO) and OLD orexin knockout (KO) and wild type (WT) mice. Values are reported as mean ± SEM. *p < 0.05 for the main effect of genotype at 2‐way ANOVA.

### 3.3 **EEG** power spectra during home‐cage recordings

**FIGURE 3:**
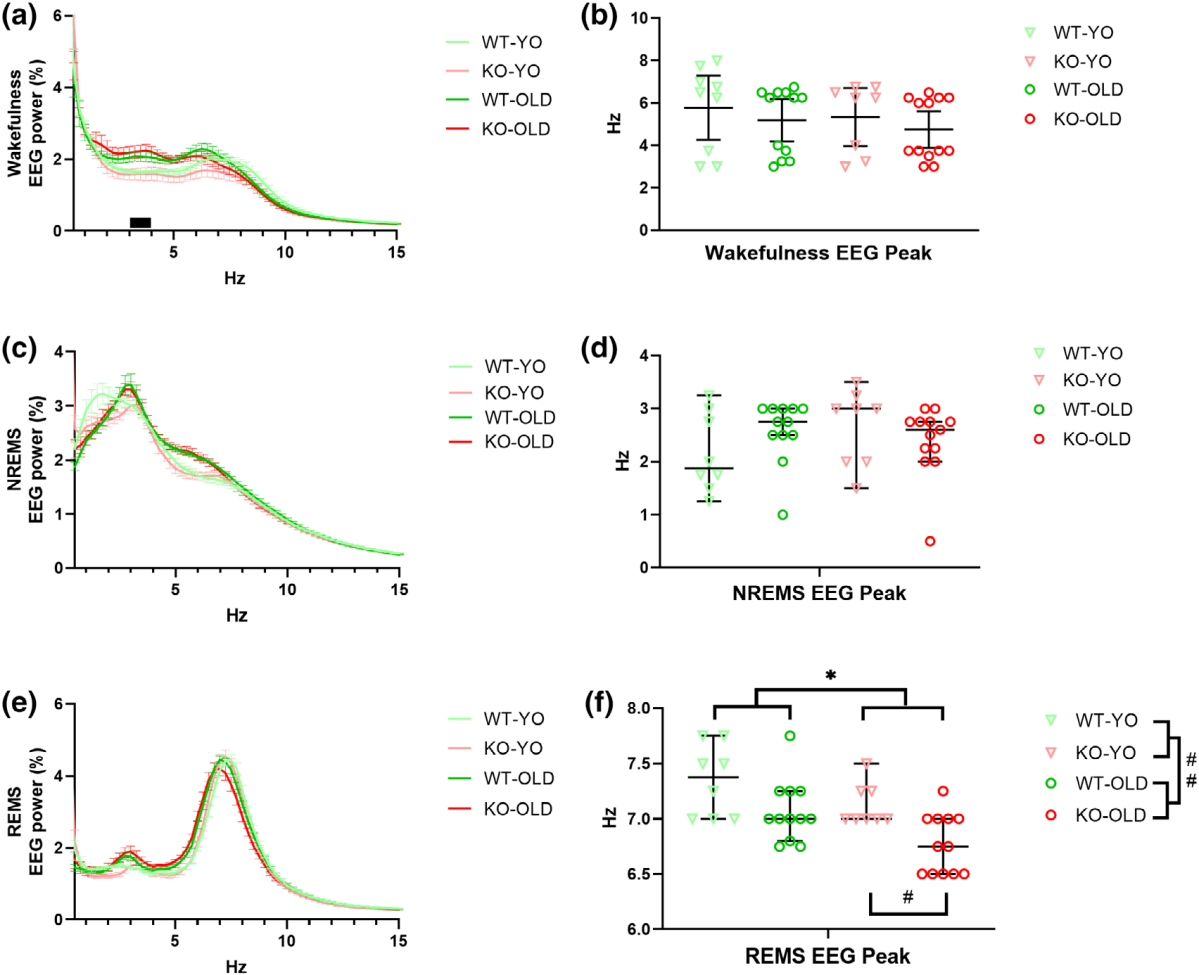
Electroencephalogram spectral analysis during wake–sleep states in home‐cage recordings. Electroencephalographic (EEG) spectral power (expressed as mean ± SEM of the percentage of total EEG spectral power) during wakefulness, non‐rapid‐eye‐movement sleep (NREMS), and rapid‐eye‐movement sleep (REMS) expressed as a percentage of total EEG spectral power (a, c, e, respectively) exhibited by OLD and young (YO) orexin‐knockout (KO) and wild‐type (WT) control mice. (b, d, f) Median (with the 95% confidence interval) values of the EEG peak frequency during wakefulness, NREMS, and REMS, with symbols indicating values in individual mice. ^#^ *p* < 0.05 for the post‐hoc corrected comparison between KO‐YO and KO‐OLD after significant Kruskal‐Wallis test. * and ^##^ genotype (*p* < 0.05) and age (*p* < 0.01) global effects, respectively, with Mann–Whitney test with the false discovery rate correction after significant Kruskal‐Wallis test. The black bar indicates *p* < 0.05 for the post‐hoc corrected comparison between OLD and YO mice, with significant age × EEG frequency interaction. KO, orexin‐knockout; WT, wild‐type; YO, young.

The EEG power spectra, the spectral peaks in any behavioural state, and the SWA during NREMS did not generally differ significantly between KO and WT (Figure 3 and Figure S1). The only exception was that the frequency of the EEG power spectrum peak during REMS (Figure 3f) was significantly lower in KO than in WT mice (post‐hoc analyses with significant Kruskal‐Wallis test).

Ageing significantly impacted on the EEG power spectra during wakefulness (Figure 3a) and on the EEG spectral peak during REMS (Figure 3f). During wakefulness, different individual mice exhibited an EEG spectral peak in the high delta or in the theta frequency bands. OLD mice showed an EEG slowing with higher EEG power during wakefulness between 3 and 4 Hz (post‐hoc analyses with significant age × EEG frequency interaction) and a significantly slower EEG spectral peak frequency during REMS than YO controls (post‐hoc analyses with significant Kruskal‐Wallis test).

**FIGURE 4:**
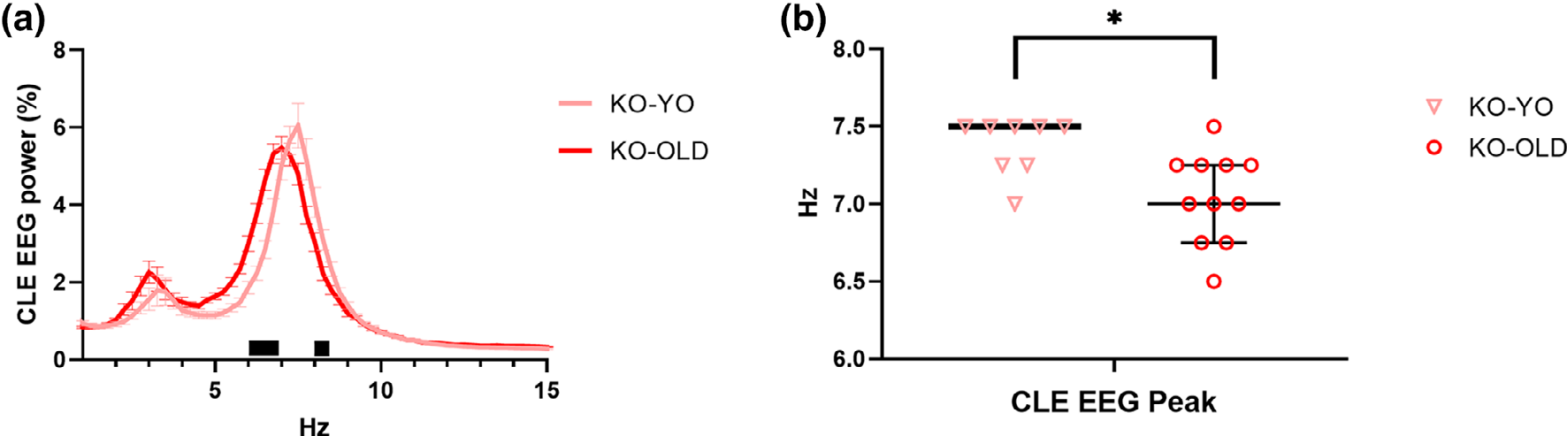
Electroencephalogram spectral analysis during cataplexy‐like episodes. Electroencephalographic (EEG) spectral power (expressed as mean ± SEM of the percentage of total EEG spectral power) and peak (median with the 95% confidence interval) during cataplexy‐like episodes (CLE) are shown for OLD and young (YO) orexin‐knockout (KO). *p < 0.05 for Mann–Whitney test with the false discovery rate correction, with significant Kruskal‐Wallis test. Black bars indicate *p* < 0.05 for the post‐hoc corrected comparison between KO‐OLD and KO‐YO mice with significant age × EEG frequency interaction.

During CLE, KO‐OLD showed significantly higher and lower EEG power than KO‐YO before and after the spectrum peak, respectively (post‐hoc analyses with significant age × EEG frequency interaction) (Figure 4a,b). Accordingly, KO‐OLD had a significantly slower EEG spectral peak frequency than KO‐YO during CLE (post‐hoc analyses with significant Kruskal‐Wallis test).

### 3.4 Breathing during sleep

**FIGURE 5:**
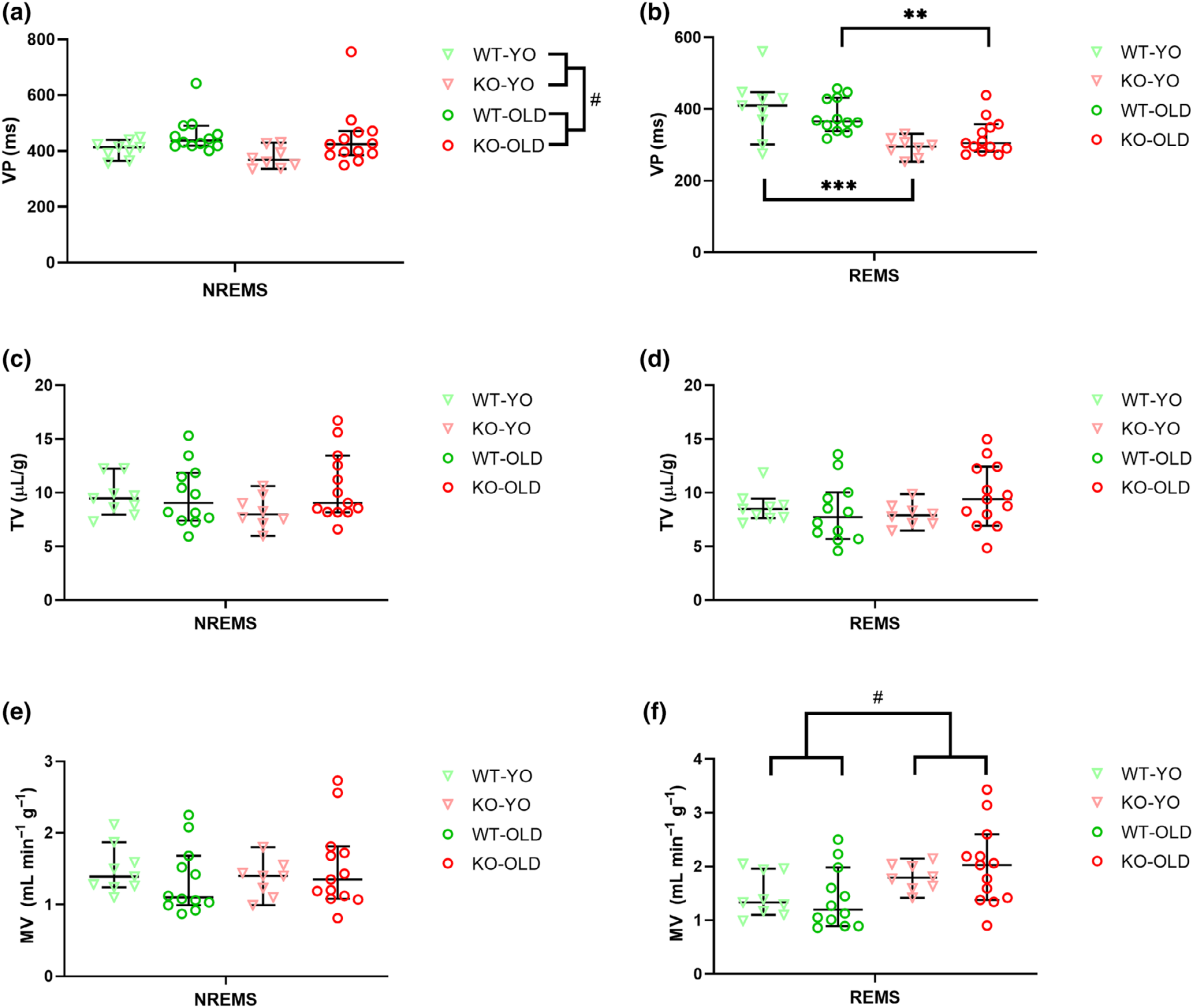
Sleep breathing phenotype. (a, b) Ventilatory period (VP) during non‐rapid‐eye‐movement sleep (NREMS) and rapid‐eye‐movement sleep (REMS), respectively, exhibited by OLD and young (YO) orexin‐knockout (KO) and wild‐type (WT) control mice. (c, d) Tidal volume (TV, corrected per body weight) during NREMS and REMS, respectively. (e, f) The minute ventilation (MV, corrected per body weight) during NREMS and REMS. Data are reported as median values (with the 95% confidence interval), with symbols indicating values in individual animals. ** and *** *p* < 0.01 and *p* < 0.001, respectively, for the post‐hoc corrected comparison between KO‐OLD and WT‐OLD and between KO‐YO and WT‐YO with significant Kruskal‐Wallis test. ^#^ *p* < 0.05 genotype or age global effect, Mann–Whitney test with the false discovery rate correction after significant Kruskal‐Wallis test. The significant age main effect is not reported in (b) to increase readability. KO, orexin‐knockout; WT, wild‐type; YO, young.

**FIGURE 6:**
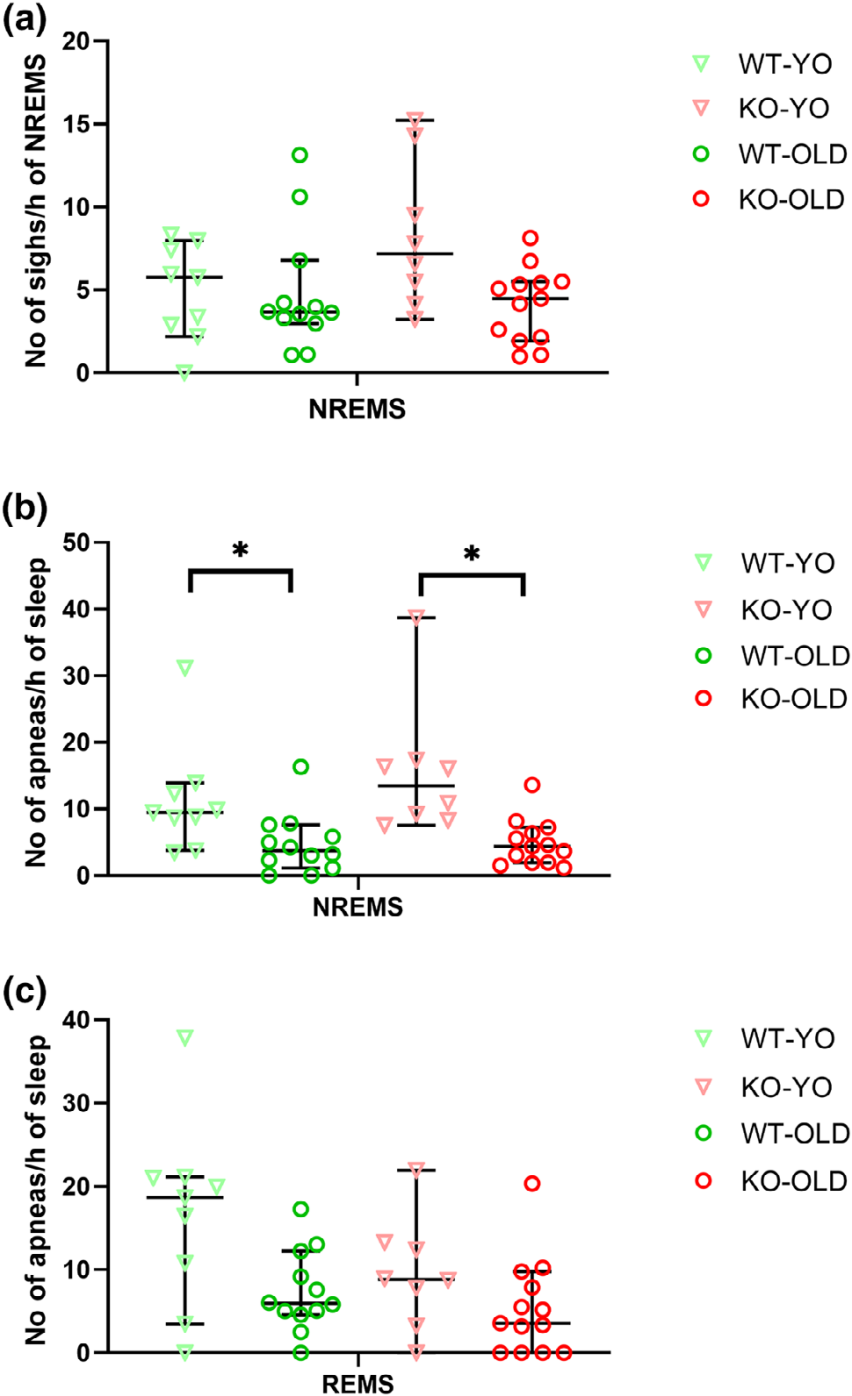
Sighs and sleep apneas. (a) Sighs (augmented breath) occurrence rate during non‐rapid‐eye‐movement sleep (NREMS) exhibited by OLD and young (YO) orexin‐knockout (KO) and wild‐type (WT) control mice. (b, c) Sleep apnea (breath pause) occurrence during NREMS and rapid‐eye‐movement sleep (REMS), respectively. Data are reported as median (with the 95% confidence interval) values, with symbols indicating results in individual mice. * *p* < 0.05, for the post‐hoc corrected comparison between WT‐YO and WT‐OLD, and between KO‐YO and KO‐OLD with significant Kruskal‐Wallis test.

Whole‐body plethysmograph recordings on the same animals that had been tested previously in their own cages provided non‐invasive information on their breathing pattern during sleep. The percentages of recording time in each wake–sleep states during the 6 h recordings inside the whole‐body plethysmograph chamber are reported in Table S1. During NREMS, none of the sleep breathing variables (VP, TV, MV, occurrence rate of sighs and apneas; Figures 5 and 6) differed significantly between WT and KO mice. The apnea occurrence rate and TV values were not affected by genotype during REMS, but VP and MV were (post‐hoc analyses with significant Kruskal‐Wallis test). Specifically, VP was significantly lower in KO‐YO than in WT‐YO and in KO‐OLD than in WT‐OLD (Figure 5b, for the sake of clarity the global significant age effect was not reported in the figure). Accordingly, MV was significantly higher during REMS in KO than in WT mice (Figure 5f).

Ageing significantly modulated VP and the sleep apnea occurrence rate during NREMS (post‐hoc analyses with significant Kruskal‐Wallis test). In particular, VP was significantly higher in OLD than YO mice (Figure 5a) while the NREMS apnea occurrence rate was significantly higher in YO than in OLD mice (Figure 6b).

### 3.5 **PCA** on **EEG** , sleep, and breathing features

**FIGURE 7:**
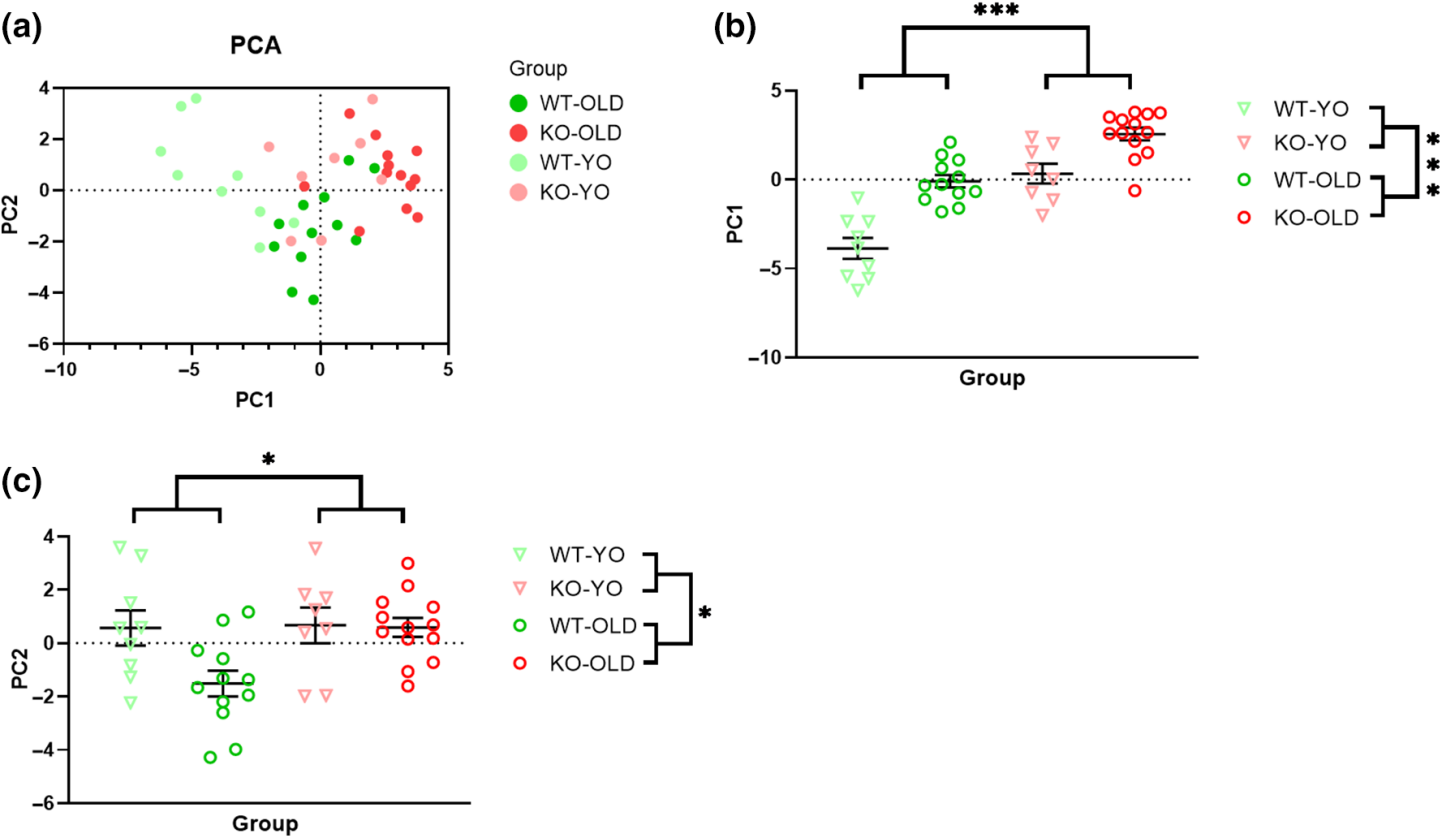
Principal components analysis. A principal components analysis (PCA) was run on 31 electroencephalographic, sleep and breathing variables of the present experiment. Scores of principal component (PC) 1 and PC2 (i.e. the two PCs explaining most of the variance in the dataset) are shown for OLD and young (YO) orexin‐knockout (KO) and wild‐type (WT) control mice. (a) Segregation of the present experimental population using PC1 and PC2 as, respectively, the x‐ and the y‐axis of a bidimensional graph. (b, c) Results of statistical comparisons between groups for PC1 and PC2, respectively. * and *** *p* < 0.05 and *p* < 0.001, respectively, for the main genotype and age effect (2‐way ANOVA).

A PCA was applied to reduce 31 features of the present experiment (EEG, sleep and breathing variables) to the two PCs (PC1 and PC2) that best explained the data variance (Figure S2). Taken together, PC1 and PC2 explained 35% of the total variance. The scores of PC1 and PC2 (i.e. t) are shown in Figure 7a while PC loadings relative to each variable (i.e. correlations between each variable and each PC) are reported in Table S2. PC1 and PC2 were both able to discriminate significantly (two‐way ANOVA, main genotype and age effects) between KO and WT, and between YO and OLD mice (Figure 7b,c).

## 4 DISCUSSION

The main finding of the present study is that the altered sleep and breathing phenotypes caused by orexin deficiency in narcoleptic mice are substantially preserved with ageing.

In line with the global picture of previous reports (Bastianini et al., 2011; Chemelli et al., 1999; Silvani et al., 2014), KO mice showed a clearcut narcoleptic phenotype, with increased NREMS time and bout number, and with reduced and fragmented wakefulness, as indicated by the increased number of episodes of shorter length, particularly during the dark (active) period (Figures 1 and 2). Moreover, KO exhibited CLE and short REMS latency (Table 1), two key features of narcolepsy in mice (Bastianini et al., 2011; Chemelli et al., 1999; Silvani et al., 2014). All these features can be visually appreciated on representative hypnograms (Figure 2). Taking account of OLD and YO mice together in this study, we found that the frequency of the EEG power spectrum peak during REMS differed between KO and WT mice (Figure 3), in contrast with what was reported previously focussing only on young‐adult KO and WT mice (Bastianini et al., 2011). As a significant slowing of the theta EEG frequency peak occurred in KO‐OLD compared with KO‐YO during both REMS and CLE (Figures 3 and 4), this discrepancy may be due to the inclusion of older mice in the present study, although the age × genotype ANOVA interaction lacked statistical significance. On the other hand, in this study, we found no significant effect of orexin deficiency (i.e. KO vs. WT) on sleep apneas (Figure 6). This is in line with our recent findings on younger mice (Berteotti et al., 2020), but contrasts with previous observations by others (Nakamura et al., 2007) that KO mice had more sleep apneas than WT.

In agreement with previous studies (Li et al., 2022; Soltani et al., 2019; Wimmer et al., 2013), we confirmed that ageing importantly modulated the mouse sleep phenotype (Figures 1 and 2). In particular, our results supported the view that OLD mice spend significantly less time awake than YO mice and exhibit higher SFI and overall wake–sleep cycle fragmentation (Li et al., 2022; Soltani et al., 2019). Our finding that ageing significantly slowed the EEG power spectrum peak during wakefulness, REMS, and CLE in mice (Figures 3 and 4) also agrees with previous data (Wimmer et al., 2013). Similarly, a significant generalised EEG slowing, affecting all frequency bands, has been reported in older human subjects (Vlahou et al., 2014). This age‐related modulation of EEG might underlie, at least in part, the increased number of epochs that were classified as undetermined (i.e. could not be confidently assigned to a specific state) in OLD than in YO.

To our knowledge, the present experiment is the first to show that in mice during NREMS, VP and sleep apnea occurrence rate, respectively, increased and decreased with age. The faster breathing pattern (lower VP) of KO during REMS compared with WT (Figure 5b) in the absence of significant changes in TV was likely causal to the significant increase in MV in KO compared with WT (Figure 5f). The increased breathing rate and MV during REMS in KO versus WT mice possibly indicated an increased energy expenditure in this sleep state. These alterations have not been observed in narcoleptic mice before. However, here we only studied female KO mice, while previous studies were performed on male mice (Berteotti et al., 2020; Nakamura et al., 2007). Male KO mice were also previously reported to be prone to obesity (Berteotti et al., 2020) while females in the present study were not. Further work should specifically investigate sex‐dependent differences in breathing during sleep in orexin‐deficient mice.

The purpose of the present experiment was to compare the sleep and breathing phenotype between KO and WT mice at different ages, to investigate whether ageing impacted on the narcoleptic phenotype. With our results and PCA analysis, we successfully replicated previous reports concerning the effects of orexin‐deficiency per se and of ageing per se on mouse sleep and breathing patterns. On the other hand, in the present experiment, we found little evidence of a significant interaction between these two factors, mainly concerning the fragmentation of wakefulness during the dark (active) period. In particular, our data indicated that WT‐YO exhibited long and consolidated episodes of wakefulness during the dark period compared with KO‐YO mice, whose mean wakefulness bout length did not differ significantly from those of KO‐OLD and WT‐OLD (Figure 2a). Recent data demonstrated that orexin neurons mildly decrease in number but become hyperexcitable in older mice, which also show fragmentation of wakefulness (Li et al., 2022). Previous work also showed that chronic ectopic hyperexpression of orexins in the brain fragments wakefulness in mice (Willie et al., 2011). Taken together, these data suggest that significant decreases in the wakefulness bout length may result from quantitative excess or from temporally inappropriate release of orexin peptides as well as from their absence.

To date, only few studies on patients with narcolepsy have investigated the influence of age on the narcolepsy traits (Furuta et al., 2001; Kovalská et al., 2016; Lividini et al., 2021; Ohayon et al., 2005). These studies are difficult given the rarity of narcolepsy and were affected by limitations such as control group matching, environmental or genetic confounders, effects of chronic treatment, and length of clinical history. These studies produced contrasting results. Some studies reported no significant differences in cataplexy with age (Kovalská et al., 2016; Ohayon et al., 2005), while others reported that older narcoleptic patients had a milder phenotype, especially in terms of cataplexy (Furuta et al., 2001; Lividini et al., 2021). A point of strength of our experiment on the KO mouse model of narcolepsy was to drastically reduce confounders and to include well‐matched control groups. In line with part of the literature on human, our data indicated that the narcoleptic sleep and breathing phenotype did not worsen or ameliorate with age compared with controls.

Our study has limitations. After 18 months of age (≈77 weeks), C57Bl6/J mice are already considered “old” (Fox et al., 2007). However, the age of our OLD groups (approximately 86 weeks) is roughly equivalent to 60–65 years in humans, which is well below the current mean human lifespan in many countries. Extrapolation of our current findings to the oldest old humans is not granted, and studies on still older narcoleptic mice are warranted. These studies should also include mouse models of orexin neuron loss, and not limit themselves to the KO model of orexin peptide deficiency, as in the present study. Our study was based on four independent groups of mice instead of longitudinal observation of the same subjects, which would have strengthened our results. Technical limitations linked to the fragility of surgical implants make this possibility challenging with gold‐standard EEG and EMG recordings. Only female mice were included in the present experiment. This allowed us to reduce experimental variability while limiting the total sample size with this complex experimental protocol and to take account of any effect of mouse reproductive senescence, as a model of human menopause (Diaz Brinton, 2012). However, sex‐dependent modulation of narcoleptic phenotype has been reported for both humans (Won et al., 2014) and mice (Arthaud et al., 2022; Piilgaard et al., 2022; Sun et al., 2022). We allowed mice 7 days to recover from surgery and 1 h to habituate to the WBP chamber before the recordings. We are aware of the potential impact on sleep of stress linked to the duration of postoperative recovery and habituation to the new environment. In mitigation, this recovery period was in line with those of similar sleep experiments by our own and other groups (Berteotti et al., 2021; Soltani et al., 2019). Moreover, prolonging habituation to the whole‐body plethysmograph would have increased the risk of damage to the head implantations due to the shorter and more rigid tether cable required by whole‐body plethysmograph recordings with respect to baseline conditions. In line with our previous works (Bastianini et al., 2011; Bastianini et al., 2021), the EEG power spectra are shown as normalised values. However, we are aware that normalisation of spectra to total power may obscure frequency‐specific differences in absolute EEG power (Panagiotou et al., 2018). Finally, it has long been known that chocolate increases the incidence of cataplectic events in murine models of narcolepsy, and therefore facilitates the study of this specific narcolepsy sign (Oishi et al., 2013). However, it cannot be excluded that the effect of this cataplectic‐promoting factor is different in young and elderly mice.

## 5 CONCLUSION

Our experiment indicated that the lack of orexins as well as ageing importantly and independently modulate sleep and breathing phenotype in female mice. The effects of orexin deficiency on the wakefulness fragmentation, and on the frequency of the EEG theta peak in REMS and CLE significantly differed between young adult and old female mice. Other features of the narcoleptic phenotype regarding sleep, breathing, and cataplexy changed less, if at all, with old age in female mice with orexin peptide deficiency.

## AUTHOR CONTRIBUTIONS

**Stefano Bastianini:** Conceptualization; investigation; methodology; formal analysis; writing – original draft; writing – review and editing. **Sara Alvente:** Investigation; writing – review and editing. **Chiara Berteotti:** Investigation; writing – review and editing. **Viviana Lo Martire:** Investigation; writing – review and editing. **Gabriele Matteoli:** Investigation; writing – review and editing. **Elena Miglioranza:** Investigation; writing – review and editing. **Alessandro Silvani:** Conceptualization; methodology; writing – review and editing; formal analysis; funding acquisition; data curation. **Giovanna Zoccoli:** Conceptualization; methodology; supervision; resources; funding acquisition; project administration; writing – review and editing; data curation.

## FUNDING INFORMATION

The research was supported by University of Bologna (RFO 2020–2022).

## CONFLICT OF INTEREST STATEMENT

The authors declare no conflicts of interest.

## Supporting information


**FIGURE S1.** Slow wave activity (SWA) is reported as electroencephalographic power in the delta frequency range (1–4 Hz) during non‐rapid‐eye‐movement sleep (NREMS) normalised to values in the last 4 h of the light period. Values are reported as 2 h bins for OLD and young (YO) orexin‐knockout (KO) and wild‐type (WT) control mice.

**FIGURE S2.** Principal component variance. Cumulative variance explained by principal components (PC) elaborated by the principal components analysis (PCA) are shown in the graph.

**TABLE S1.** Percentage of recording time in each wake–sleep state inside the whole‐body plethysmograph.

**TABLE S2.** Principal component loadings.

## Data Availability

The data that support the findings of this study are available from the corresponding author upon reasonable request.
