# Supplementary figures and images for: Ageing‐related modification of sleep and breathing in orexin‐knockout narcoleptic mice
Source: J Sleep Res. 2024 Jul 20;34(2):e14287. doi: 10.1111/jsr.14287 (PMC11911059; doi:10.1111/jsr.14287)

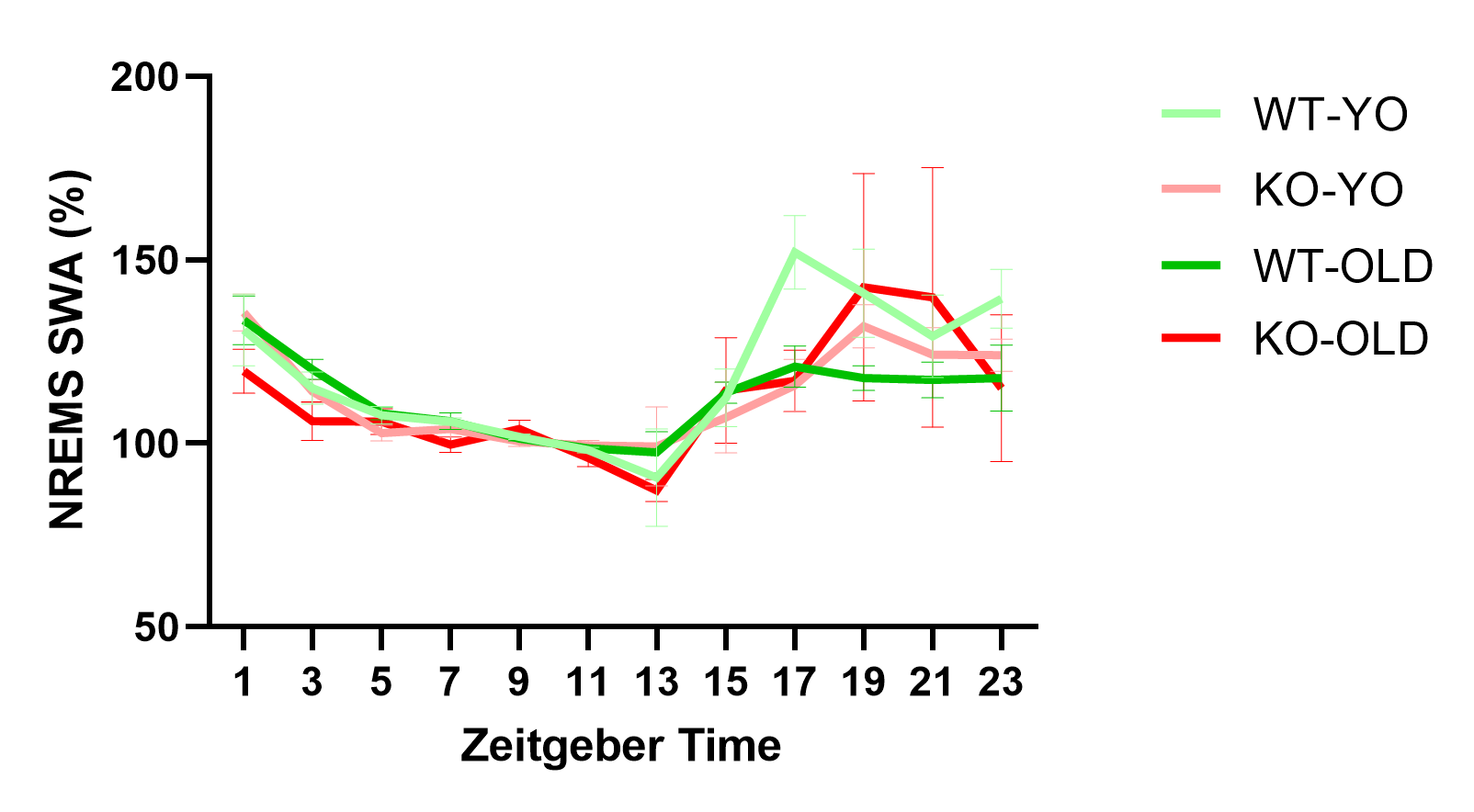

Supplement: Supplementary file 1 — FIGURE S1. Slow wave activity (SWA) is reported as electroencephalographic power in the delta frequency range (1–4 Hz) during non‐rapid‐eye‐movement sleep (NREMS) normalised to values in the last 4 h of the light period. Values are reported as 2 h bins for OLD and young (YO) orexin‐knockout (KO) and wild‐type (WT) control mice. [file JSR-34-e14287-s003.tif]

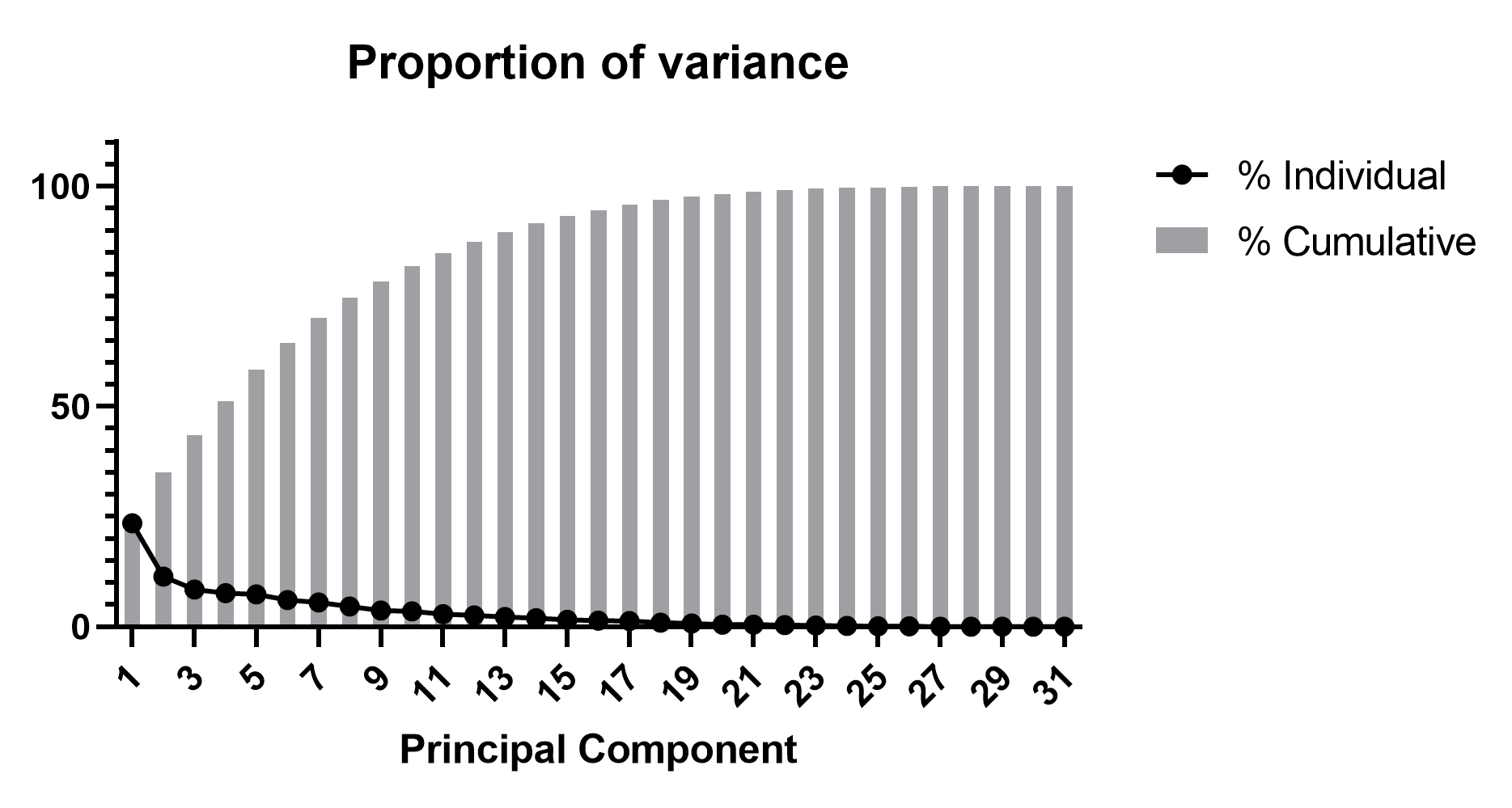

Supplement: Supplementary file 2 — FIGURE S2. Principal component variance. Cumulative variance explained by principal components (PC) elaborated by the principal components analysis (PCA) are shown in the graph. [file JSR-34-e14287-s002.tif]
