# Supplementary material for: Ageing‐related modification of sleep and breathing in orexin‐knockout narcoleptic mice
Source: J Sleep Res. 2024 Jul 20;34(2):e14287. doi: 10.1111/jsr.14287 (PMC11911059; doi:10.1111/jsr.14287)
Supplement: Supplementary file 3 — TABLE S1. Percentage of recording time in each wake–sleep state inside the whole‐body plethysmograph. [file JSR-34-e14287-s001.docx]

**Table S1. Percentage of recording time in each wake-sleep state inside the whole-body plethysmograph**

|  | **Wakefulness (%)** | **NREMS (%)** | **REMS (%)** |
| --- | --- | --- | --- |
| **WT-YO** | 28.5 (37.4) | 62.6 (34.7) | 6.7 (9.7) |
| **KO-YO** | 22.5 (24.9) | 66.7 (22.7) | 7.5 (3.7) |
| **WT-OLD** | 22.2 (28.4) | 61.7 (28.7) | 7.9 (5.3) |
| **KO-OLD** | 24.8 (20.8) | 67.8 (19.1) | 4.7 (7.4) * |

Percentage of recording time during wakefulness, non-rapid-eye-movement sleep (NREMS), and rapid-eye-movement sleep (REMS) in OLD and young (YO) orexin-knockout (KO) and wild-type (WT) control mice during the 6-h recordings inside the whole-body plethysmographic (WBP) chamber. Data are reported as median (range). *, P < 0.05 vs. WT-OLD (post-hoc analyses after significant Kruskal-Wallis test).
