# Supplementary material for: Ageing‐related modification of sleep and breathing in orexin‐knockout narcoleptic mice
Source: J Sleep Res. 2024 Jul 20;34(2):e14287. doi: 10.1111/jsr.14287 (PMC11911059; doi:10.1111/jsr.14287)
Supplement: Supplementary file 4 — TABLE S2. Principal component loadings. [file JSR-34-e14287-s004.docx]

**Table S2. Principal Component Loadings**

| **Var** | **PC1** | **PC2** |
| --- | --- | --- |
| TV N | 0.10782 | -0.36335 |
| TV R | -0.01797 | -0.39709 |
| VP N | 0.294479 | 0.074235 |
| VP R | 0.384578 | 0.371463 |
| Apnea N | -0.55943 | 0.518426 |
| Sigh N | -0.32593 | 0.251576 |
| Apnea R | -0.06895 | 0.394422 |
| freq W light | 0.562697 | -0.39601 |
| duration W light | -0.73203 | 0.124651 |
| freq W dark | 0.809872 | -0.12257 |
| duration W dark | -0.7588 | 0.266558 |
| freq N light | 0.454249 | 0.415439 |
| duration N light | -0.24875 | -0.07442 |
| freq N dark | 0.686246 | 0.14346 |
| duration N dark | -0.60718 | -0.20005 |
| %W light | -0.27025 | -0.53511 |
| %W dark | -0.81388 | -0.18309 |
| %N light | 0.027105 | 0.628 |
| %N dark | 0.673085 | 0.226829 |
| %R light | 0.175009 | -0.22546 |
| %R dark | 0.522911 | -0.12223 |
| freq R light | 0.566651 | 0.111802 |
| dur R light | -0.59186 | 0.425518 |
| freq R light | 0.665261 | -0.07217 |
| freq R dark | -0.35169 | 0.627233 |
| R latency light | -0.21039 | -0.4457 |
| R latency dark | -0.56299 | -0.4683 |
| CLE | 0.42782 | 0.194761 |
| Peak W | -0.1157 | -0.4636 |
| Peak N | 0.068829 | 0.0278 |
| Peak R | -0.38178 | -0.14632 |

The table shows the correlation coefficients between each of the 31 features of the present experiment (Var) and the 2 Principal Components (PC) explaining the highest variance selected with the principal component analysis (PCA). The coefficients higher than 0.7 or lower than -0.7 are highlighted in green for ease of visualization.
